# Revisiting the effect of colonial institutions on comparative economic development

**DOI:** 10.1371/journal.pone.0177100

**Published:** 2017-05-08

**Authors:** Valentina A. Assenova, Matthew Regele

**Affiliations:** Management Department, Yale University, New Haven, CT, 06520, United States of America; Tianjin University of Technology, CHINA

## Abstract

European settler mortality has been proposed as an instrument to predict the causal effect of colonial institutions on differences in economic development. We examine the relationship between mortality, temperature, and economic development in former European colonies in Asia, Africa, and the Americas. We find that (i) European settler mortality rates increased with regional temperatures and (ii) economic output decreased with regional temperatures. Conditioning on the continent of settlement and accounting for colonies that were not independent as of 1900 undermines the causal effect of colonial institutions on comparative economic development. Our findings run counter to the institutions hypothesis of economic development, showing instead that geography affected both historic mortality rates and present-day economic output.

## Introduction

A longstanding debate in comparative politics and political economy has centered on the reasons for the large observed differences in economic output across states. Advocates of the geography hypothesis posit that differences in regional temperatures determined states’ paths to economic growth through their effect on the prevalence of diseases [1–3]. In this view, states with warmer climates and higher average annual temperatures experienced larger disease prevalence than states with cooler climates [4–6]. Disease burdens in turn produced poverty traps whereby low levels of human development decreased the quality of political and legal institutions [7–9]. This perspective has called for stimulating economic development primarily through the provision of foreign aid.

By contrast, advocates of the institutions hypothesis have argued that the quality of legal and political institutions across states—rather than differences in geography—produced divergent paths of economic growth. Institutional quality affected states’ economic development trajectories by influencing their rates of investment and technological innovation [10, 11]. According to this perspective, the different economic development paths of former European colonies in Asia, Africa, and the Americas arose from the quality of the institutions established by European colonialists [12, 13]. As the argument goes, where Europeans faced high settler mortality rates, they established extractive institutions—institutions that encouraged Europeans to exploit natural resources and labor—that ultimately produced poor economic growth and low development [12, 14, 15]. Meanwhile, where European colonialists faced low mortality rates, they established inclusive institutions that promoted democracy and fair distribution [10, 11]. Colonies with extractive institutions—in present-day states such as as Nigeria and Mali—experienced persistent poverty. By contrast, colonies with inclusive institutions—such as the United States and Canada—prospered over time [12, 14, 15].

Persistent poverty and stagnant economic growth in former European colonies as varied as the United States, India, and Nigeria have thus been attributed to the extractive institutions established by Europeans rather than to geographically determined disease burdens [10, 11, 14–17]. Research adopting this view of comparative development has postulated a negative relationship between European settler mortality rates and the quality of historical political institutions, such as democracy and the constraint on the executive in 1900. Empirical studies that have used settler mortality as an instrument for the quality of historical institutions have also claimed that European settler mortality affected contemporary differences in economic growth by affecting the quality of these institutions [10–12]. The use of the mortality instrument has thus been purported to yield causal estimates of the effect of institutional quality on differences in economic output among former European colonies in Africa, Asia, and the Americas.

We examine the usefulness of the settler mortality measure as an instrument for the quality of historical political institutions in the sample of former European colonies used to establish the causal relationship [12]. Our analyses demonstrate that the mortality measure is highly sensitive to the method of imputation for 43 of the 64 former European colonies that were not independent states as of 1900. After changing the values of the key measure of the quality of historical political institutions, which was imputed in the original data for all colonies in Africa, settler mortality fails to predict differences in the quality of colonial institutions at the first stage of the two-stage least squares estimation procedure used to estimate its causal effect on economic development. We also find that increases in mean temperature predict increases in historical European settler mortality rates and decreases in economic output. Conditioning on the continent of settlement, settler mortality fails to predict differences in the quality of historical political institutions.

Our findings demonstrate the importance of geography as a determinant of settler mortality rates, the quality of colonial institutions, and differences in economic output in former European colonies. The evidence that we present about the direct relationship between geography, historic settler mortality rates, and economic output runs counter to the institutions hypothesis of economic development [10–12]. Our results remain robust to different measures of institutional quality and economic output, as well as to the inclusion of a potential omitted variable that explains differences in economic development: institutional age. We discuss the implications of our findings for research on the geographic and institutional determinants of comparative-historical economic development and conclude with directions for future research.

## Materials and methods

We proceed with our analyses by first examining the relationship between European settler mortality and mean temperatures within regions—a proxy for countries’ disease burdens [1, 2, 6]. The data and replication code for our analyses are publicly available through the Harvard Dataverse: doi:10.7910/DVN/FP8THH. Many prior studies have noted the role of geography in disease burdens and the quality of political institutions [3, 5–7]. We therefore examine whether mean regional temperature directly predict historical rates of European settler mortality and present-day differences in economic output, thus undermining the purported causal relationship between colonial institutions and development [10–12].

We then turn to an analysis of the original data used to establish the validity of the European settler mortality instrument [9, 12] and explore whether the assumptions necessary to establish a causal relationship between colonial institutions and comparative development in former colonies are validated. Two-stage least squares estimation using an instrumental variable requires that the instrument (European settler mortality) is assigned exogenously or “as-if randomly” across the units of analysis (colonies) with respect to the dependent variable of interest (economic output) [18, 19]. For the results and the causal effects to be valid, the instrument should not be connected to the dependent variable in any way other than through the proposed causal path, which is the quality of historical political institutions established by Europeans. These assumptions mean that there should be no correlations (including through other mediating variables) between settler mortality and per capita economic output.

We test both the exclusion restriction of the instrument conditional on past institutions and the strength of the instrument for explaining within-continent variation in historical political institutions [18, 19]. We then explore the sensitivity of the predictive power of the instrument to the imputation of the missing values for institutional quality among the states that were not independent as of 1900 (67% of the sample in the original study) through simulations using two-stage least squares instrumental variable regressions [12].

## Results and discussion

In the tables and figures below, we present evidence that the exclusion restriction and “as-if random” assignment of the European settler mortality instrument may both be violated, thus undermining the validity of the instrument and the purported causal relationship between colonial institutions and comparative economic development [10–12, 20]. We present evidence of a direct relationship between (i) mean temperature (in Celsius) across regions and differences in historical rates of settler mortality (deaths per 1,000 soldiers) and (ii) between mean temperature and current levels of economic output (GDP). These results lend evidence that geographic factors affected present-day differences in comparative development across former European colonies, through both disease burdens [3, 5, 6, 8] and institutional quality [4, 7, 9, 15].

### Effect of temperature on mortality and economic output

**Fig 1:**
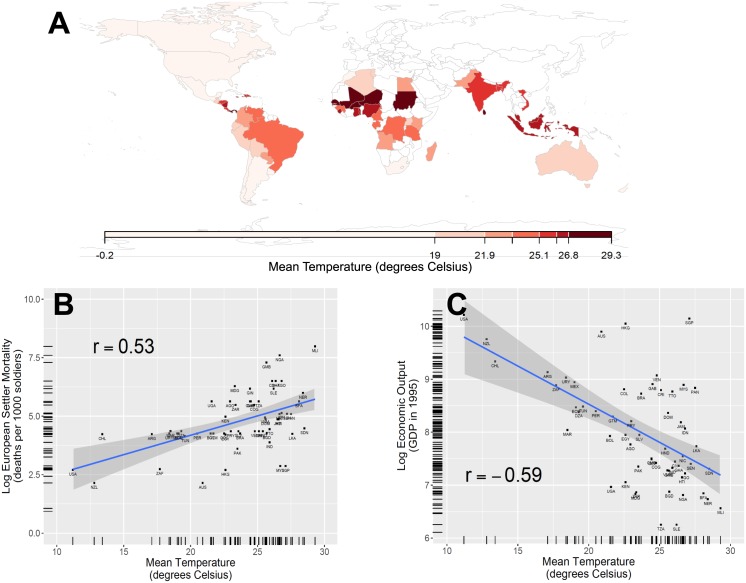
Mean temperature, European settler mortality, and economic output per capita in former European colonies. A: Map showing the mean annual temperatures (in degrees Celsius) in the former European colonies in the Americas, Africa, and Asia. B: OLS regression fit with 95% confidence intervals for the effect of mean temperature on logged European settler mortality rate, and the bivariate Pearson correlation (inset). C: OLS regression fit with 95% confidence intervals for the effect of mean temperature on economic output per capita, and the bivariate Pearson correlation (inset).

Fig 1 plots the distributions of mean annual temperatures (in degrees Celsius) in the former European colonies—present-day states in Africa, Asia, and the Americas (Panel A), and the partial correlations between these temperatures and the historic mortality rates of European settlers in these areas (Panel B) and between these temperatures and present-day economic output per capita (Panel C). We find that both historical European settler mortality rates and present-day economic output are strongly positively correlated with increases in mean temperature across regions (Pearson correlation = 0.53 and -0.59, respectively). The bivariate relationship between European settler mortality [12] and mean temperature [21] shows that increases in mean temperatures are associated with spikes in mortality rates, consistent with geographic disease burden explanations for differences in development. Furthermore, the bivariate relationship between mean temperatures and economic output per capita shows that increases in temperature were associated with lower economic output in former European colonies [3, 7, 21].

Table 1 presents the coefficients from regression models of the effect of mean temperatures on historic settler mortality rates, economic output per capita, and institutional quality. We used two measures of institutional quality established in the literature: the constraint on the executive in 1900 and the average protection against expropriation risk between 1985 and 1995 [12]. The positive coefficient of Mean Temperatures (*β* = 30.30, p<0.05) confirms that every one degree (Celsius) increase in mean temperatures was associated with an increase in European settler mortality of 30 deaths per 1,000 soldiers. Further, the negative coefficient of Mean Temperature (*β* = -0.12, p<0.01) confirms that every one degree (Celsius) increase in mean temperatures was associated with a 12 percent decrease in per capita economic output (Log GDP per capita in 1995). We find similar negative effects of increases in mean temperatures on the quality of political institutions as measured by the constraint on the executive in 1900, with increases in temperature predicting lower constraint on the executive (*β* = -0.25, p<0.01) and lower average protection against expropriation risk between 1985 and 1995 (*β* = -0.15, p<0.01).

**Table 1:** Effect of mean temperature on settler mortality, economic output per capita, and the quality of political institutions.

	*Dependent variable:*			
	Settler Mortality	Log GDP per capita, 1995	Constraint on Executive	Protection against Expropriation
Mean Temperature	30.30**	−0.12***	−0.25***	−0.15***
	(12.72)	(0.02)	(0.04)	(0.03)
Constant	−434.40	10.85***	7.90***	9.96***
	(302.46)	(0.52)	(1.02)	(0.82)
*N*	57	60	58	60
*R*^2^	0.26	0.55	0.48	0.36

* p<0.1;

** p<0.05;

*** p<0.01

All regressions are OLS. Standard errors are in parentheses. Constraint on executive in 1900 is on a scale from 1 to 7, with a higher score indicating greater constraint. Protection against expropriation risk (on an index of 0 to 10 where a higher score implies a higher protection) denotes the quality of political institutions at protecting property rights.

These results show that differences in geography are directly associated with differences in the quality of historical institutions (the constraint on the executive in 1900), present-day political institutions (average protection against expropriation risk between 1985 and 1995), and present-day economic output (Log GDP per capita in 1995). Our results therefore appear to support the presence of a direct link between geography, disease burdens, and institutional quality.

### Exclusion restriction

To understand whether colonial institutions had a direct causal effect on differences in present-day economic output, we tested the validity of the assumptions behind the two-stage least squares instrumental variable models used in the original study to establish the causal relationship [12]. Table 2 presents coefficient point estimates from linear regression models of the direct effect of European settler mortality on differences in economic output (Log GDP in 1995, Log GDP per Worker, and Log GDP in 2012). For European settler mortality to be a valid instrument of historical institutions, it must not have a direct effect on current differences in economic output other than through its effect on the quality of historical institutions established by European settlers (“exclusion restriction”) [19].

**Table 2:** Test of the exclusion restriction of the effect of past institutions on economic output per capita.

	*Dependent variable:*			
	Log GDP 1995	Log GDP/Worker	Log GDP 2012	
Log European Settler Mortality	−0.45***	−0.53***	−0.47***	−0.46***
	(0.09)	(0.11)	(0.10)	(0.10)
Institutional Age in 1817			0.002***	
			(0.001)	
Institutional Age in 1900				0.002***
				(0.001)
Constraint on the Executive in 1900	0.08	0.08	0.07	0.07
	(0.05)	(0.06)	(0.06)	(0.06)
Constant	9.97***	10.91***	10.31***	10.15***
	(0.52)	(0.59)	(0.59)	(0.60)
*N*	60	57	57	57
*R*^2^	0.46	0.48	0.55	0.56

* p<0.1;

** p<0.05;

*** p<0.01

All regressions are OLS. Standard errors are in parentheses. Constraint on executive in 1900 is on a scale from 1 to 7, with a higher score indicating greater constraint. Institutional age is years since the first European colonialization until 1817 and 1900, respectively.

We find that there is a strong, direct negative partial correlation between settler mortality and economic output, as shown in Table 2. The presence of a direct relationship between European settler mortality and present-day economic output appears to violate the exclusion restriction for the instrument. The negative regression coefficients for Log European Settler Mortality confirm a statistically significant relationship between increases in settler mortality and decreases in economic output. Indeed, every one percent increase in the historical rate of settler mortality within a former colony was associated with a 0.45 percent decrease in economic output in 1995 (*β* = -0.45, p<0.01), a 0.53 percent decrease in the economic output per worker (*β* = -0.53, p<0.01), and a 0.47 percent decrease in economic output in 2012 (*β* = -0.47, p<0.01). Controlling for past institutions and regressing the log settler mortality directly on current economic performance produced significant negative results, meaning that European settler mortality directly predicted present-day differences in economic output. This evidence is consistent with a violation of the exclusion restriction of the mortality instrument, since there should be no direct effect of the instrument on the dependent variable or any effect running through omitted variables [19].

### Institutional age and disease immunity

The institutions that were established under European colonial rule in Asia, Africa, and the Americas would have begun to develop when Europeans first colonized the regions under analysis [15]. The timing of institutional formation may thus confound institutional development with other age-dependent factors, such as the growth in the size of European settlements, the evolving reputation of settlers and its impact on the strength of extractive institutions, and the density of the European population in the colonies. Prior research has shown, for example, that agents’ reputation over time contributes to the level of cooperation within a group in spatial public goods games [22]. Colonies with older institutions may therefore have had more time over which European settlers developed reputations as colonialists among indigenous people, thereby influencing cooperation about how to distribute local resources. More cooperative societies may have been more prone to establish inclusive—rather than extractive—political institutions [14].

Additionally, European settler mortality might also be partially determined by institutional age, as Europeans living in a colony that was established in 1500 might have developed immunity to local diseases by the time that the mortality rates were beginning to be collected in 1817. There is considerable heterogeneity in the dates when each of the colonies included in the base colony and ex-colony samples were first colonized by Europeans, specifically with respect to some of the present-day African states, which were colonized as late as the early 20th century and some of the present-day South American states, which were colonized as early as the late 15th century. Susceptible-infective-recovered (SIR) models of the spread of diseases show that increases in immunity improve recovery rates and lower future susceptibility to diseases, thereby limiting mortality rates over time [23, 24]. Thus, the longer that Europeans had colonized a region, the more likely it is that they developed immunity to local diseases. Institutional age should thus affect settler mortality rates.

To examine such possibilities, we constructed an institutional age variable by coding the dates for the earliest documented European settlement [25]. We then subtracted the date of settlement from the dates that were important to the original analysis, namely 1817 and 1900. We found that institutional age explained variation in present-day economic output. Institutional age at the time of the mortality measurement indeed predicts lower settler mortality rates observed in 1817. The negative relationship is consistent with the idea that settlers in older colonies developed immunity to local pathogens by the time that the mortality data on solider deaths were collected. Institutional age also predicts differences in present-day economic output. Specifically, older institutions are correlated with higher economic output, possibly through the effect of institutional stability on economic growth [10].

### Non-random assignment of mortality to regions

**Fig 2:**
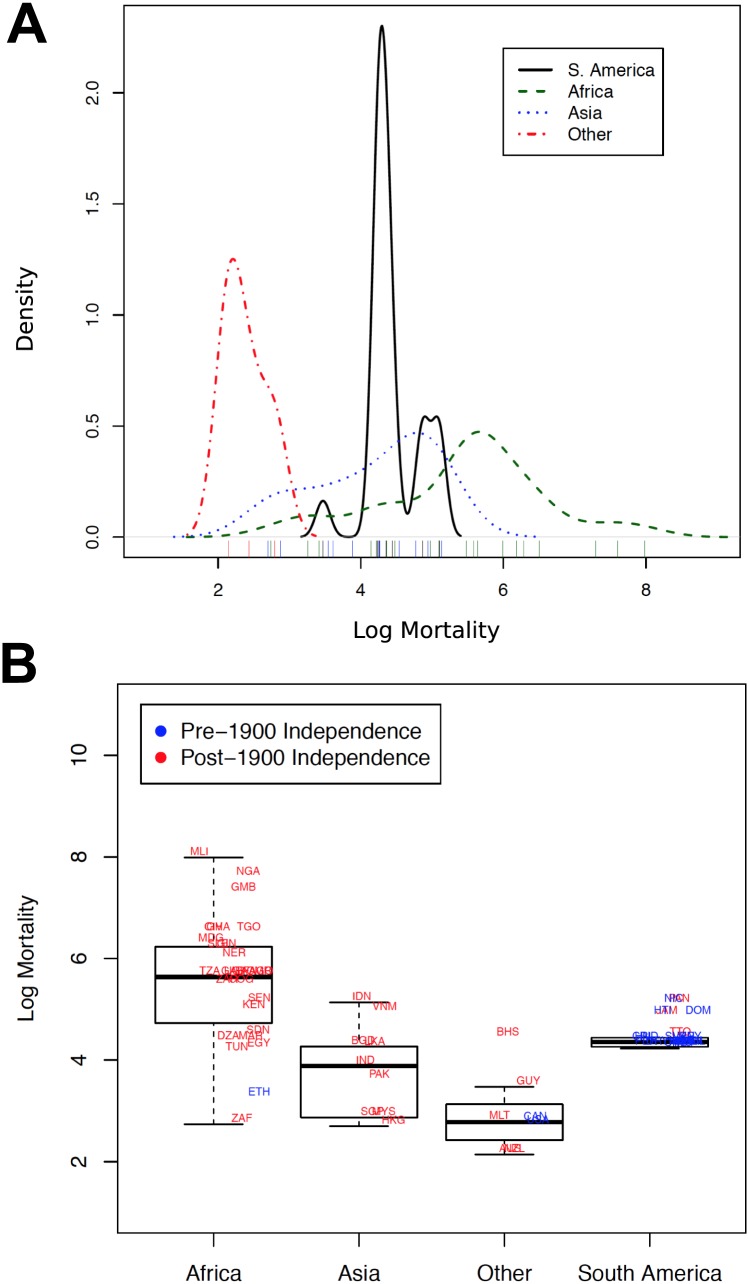
European settler mortality by continent. A: Density of the logged mortality measure by continent. B: Box plots of the mean and interquartile ranges of the logged mortality measure by continent.

A second requirement for European settler mortality to be a valid instrument of the quality of historical political institutions is that mortality needs to have been as-if randomly assigned to former European colonies. Fig 2 shows the density of log mortality by continent. From the density plots, it is immediately obvious that the mortality measure is not randomly assigned to colonies within continents. We find that low settler mortality rates were driven largely by “other” countries not in the South American, African, or Asian colonies. This group comprises today’s high income states, including the United States, Canada, and Australia. By contrast, the colonies with the highest settler mortality rates are dis-proportionally African.

Of additional concern is the large, narrow spike we see for South America. This spike is a somewhat concerning given the fact that most of the original data for South America come from an entirely different dataset and base population (Catholic cardinals) than the rest of the data (which are based on soldier mortality). The markedly different shapes of these distributions suggest that the datasets might not be comparable. The large spike in the South America data also raises the question of whether there is sufficient variation in the measure to make it useful. At the least, the density plots of log mortality by continent raise the question of whether the instrument was plausibly as-if randomly assigned to European colonies in different geographic regions. The same question arises if we examine the box plot in Fig 2 (Panel B). Here we see close clustering of colonies within continents, and almost no variation in the mortality data for South American colonies. Such patterns appear consistent with the geography hypothesis of comparative development [1, 3, 6, 9]. If the mortality instrument was randomly assigned to colonies, however, we should be able to replicate the substantive results even after examining the relationship between settler mortality and economic output across colonies within the same continent.

### Failure of the instrument to predict differences in institutional quality within regions

We proceed to split the sample by continent and test the predictive power of the European settler mortality instrument for determining differences in the quality of past institutions among former colonies. If colonial institutions determined differences in present-day economic output in former colonies, we should observe that settler mortality predicts differences in economic output through its effect on the quality of political institutions [10–12].

Table 3 reports coefficient point estimates from linear regression models predicting differences in the quality of present-day political institutions (average protection against the risk of expropriation between 1985 and 1995) from variation in the logged European settler mortality rates (deaths per 1,000 soldiers in 1817). As none of the coefficient estimates of the effect of Log European Settler Mortality are statistically different from zero, settler mortality does not appear to be a predictor of the average risk of expropriation at the first stage of the estimation. Excluding the North American colonies (the present-day United States and Canada), the partial correlation between settler mortality and the quality of contemporary political institutions in the African, Asian, and South American colonies is statistically indistinguishable from zero. We attribute the insignificant partial correlation between mortality and institutional quality to the fact that there is almost no variation in the past institutions measure within Asia and Africa, and almost no variation in the mortality measure within South America.

**Table 3:** Effect of European settler mortality on average protection against expropriation risk.

	*Dependent variable:*		
	Protection against Expropriation, 1985-1995		
	S. America	Africa	Asia
Log European Settler Mortality	−0.76	−0.11	−0.81
	(0.65)	(0.20)	(0.48)
Constant	9.77***	6.48***	10.33***
	(2.88)	(1.12)	(1.90)
*N*	23	27	9
*R*^2^	0.06	0.01	0.29

* p<0.1;

** p<0.05;

*** p<0.01

Protection against expropriation risk (on an index of 0 to 10 where a higher score implies a higher protection) denotes the quality of political institutions at protecting property rights.

Table 3 shows that the negative relationship between mortality and the average protection against expropriation risk does not hold when we examine differences in economic output across colonies within the same continent. The claim that lower settler mortality contributed to higher quality institutions (higher average protection against expropriation risk) implies that we should observe that as mortality decreases, institutional quality increases. However, we do not see this pattern in general in the data or in the split-sample results. Our coefficient estimates for the models predicting the effect of mortality on the average protection against expropriation for the Asian, African, and South American colonies (columns 2, 3, and 4 in Table 3) are not statistically significantly different from zero (p>0.10). These results show that the relationship between the instrument (settler mortality) and the quality of political institutions does not hold in the split sample at the first stage of the two-stage least-squares estimation.

## Sensitivity to imputation

Historical data on various indicators of institutional quality and human capital are often difficult to collect systematically, especially when one is talking about spans of hundreds of years and regions across the globe. The original data used to validate the settler mortality instrument had many missing values for former European colonies in the African continent, requiring various assumptions to enable data imputation [12]. The variables used in these analyses as proxies for the quality of historical institutions—the constraint on the executive and the democracy indicator—were both sourced from historical records dating to 1900, when all of the African colonies (about 67% of the sample) were not yet independent. Thus, the study that validated the instrument relied on assigning the lowest value of the quality of political institutions for missing values in the African colonies [12].

We explore the sensitivity of the first stage instrumental variable regression results to the assignment of the minimum values for all colonies in Africa and other colonies that were not independent as of 1900. We show that under simulations of the causal effect of colonial institutions on economic output, the p-values for the log European settler mortality are insignificant in the majority of simulations imputing the quality of political institutions (protection against expropriation risk between 1985 and 1995, and the constraint on the executive in 1900). Indeed, the European settler mortality instrument does not seem to predict the quality of past institutions (constraint on the executive in 1900) in the first stage of the two-stage least squares instrumental variable regressions under different missing data imputation assumptions.

The reliance on measures from 1900 also presents a few challenges with the analyses, the most important of which is the fact that for both of the measures of political institutions, data are missing for a large number of states that remained colonies in 1900. The study that first used the mortality instrument overcome this challenge by assigning the lowest possible value (1 for constraint on the executive and 0 for democracy) to these cases [12]. This imputation of missing cases greatly skewed the data towards supporting the institutional hypothesis of economic development [10–12]. The assumption also seems unrealistic.

**Fig 3:**
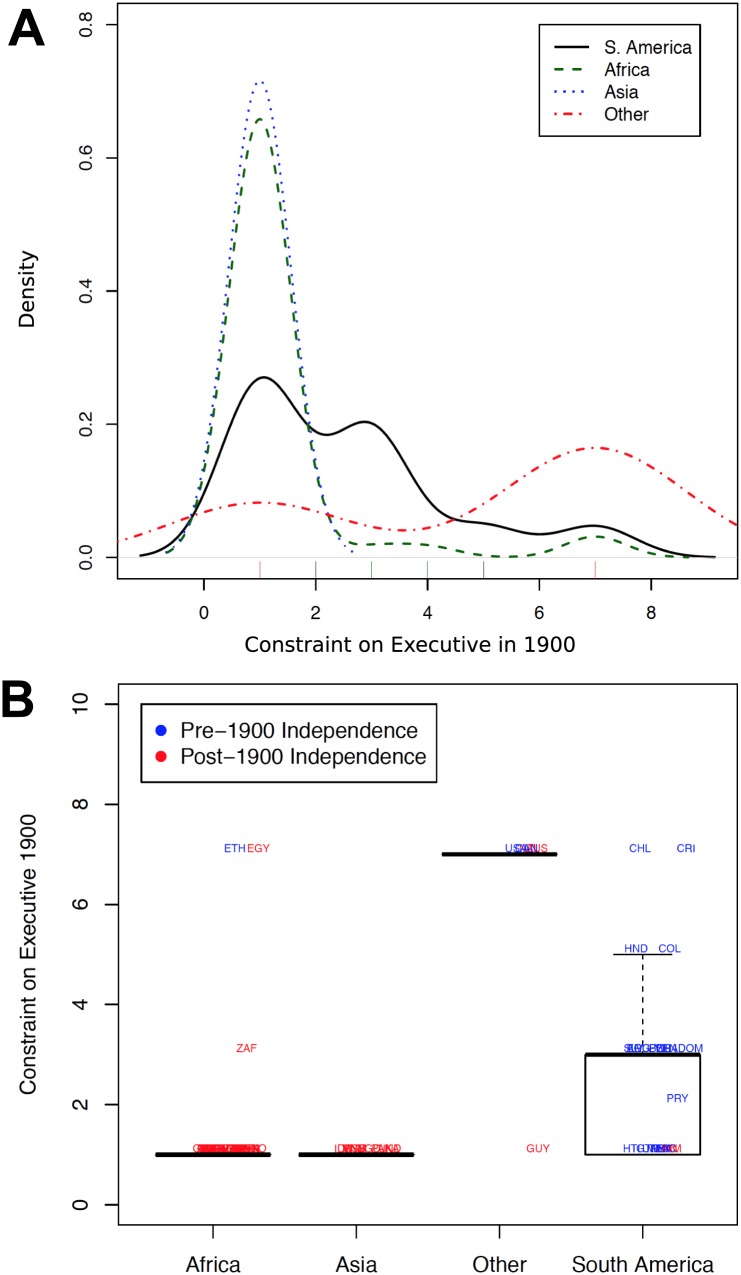
Constraint on the executive in 1900 by continent. A: Density of the constraint on the executive in 1900 measure by continent. B: Box plots of the mean and interquartile ranges of the constraint on the executive measure by continent.

Consider the United States. By the imputation methods above, if the data about the American colonies came from 1787 rather than 1900, the authors of the original study should have assigned the United States an institutional quality score of zero or one. Knowing the history of the American colonies, however, this imputation seems unreasonable. Although the colonies were nominally controlled by the British government prior to independence, they also enjoyed comparatively high political freedom and democratic self-rule. Further, even if the American colonies were completely ruled by Britain, the “executive” would have been the king, who did not rule free from constraint, since Parliament greatly influenced his actions. Although the United States represents an extreme case, it seems plausible that many colonies may have enjoyed at least some self-rule and democracy in 1900, and their executive likely faced some constraint. If this is the case, the original study used to establish a causal connection between the quality of colonial institutions and current differences in economic output [12] is likely imputing missing values of the data to the benefit of establishing a causal effect. Data imputation of the missing values for the quality of historical institutions may be especially problematic given that the vast majority of the missing cases are from African colonies. Fig 3 shows the distribution of the constraint on executive measure and demonstrates how concentrated this measure is on the lower end of the scale for the African and Asian colonies.

Although we are uncomfortable with assigning the lowest values for all of the missing cases, we are also unsure of the best alternative. Given the small sample size, it is difficult to think of any convincing way to systematically impute missing values. The authors of the original study do not report to which countries they assigned minimum values for these variables. Indeed, they do not indicate what fraction of their base sample remained dependent colonies as of 1900. As a result, it is difficult to conduct sensitivity checks to see how the imputation assumptions affect the validity of the European settler mortality instrument and of the original results. Nevertheless, since we saw the data imputation issues as creating a particularly large bias, we did our best to conduct such an analysis.

Our first step was to try and determine which European colonies had not yet achieved independence in 1900. As our data source, we relied on the Central Intelligence Agency’s “World Factbook”, which contains an online listing of state independence dates. According to the World Factbook, 43 of the 64 colonies (67%) in the base sample were not independent in 1900. Of these colonies, over half (26) were located in Africa. Interestingly, despite the colonies’ post-1900 independence dates, the original data do not seem to be missing values for all of these cases, as 8 of the 43 do not have values of 1 entered for constraint on the executive in 1900. Of these eight, Australia, Egypt, and New Zealand have values of 7 and South Africa has a value of 3. The other cases (The Bahamas, Hong Kong, Malta, and Sierra Leone) have missing values for the constraint on the executive. Thus, we are left with the fact that the data may have been imputed for roughly half of the original sample. The assumptions made for this imputation seem aggressive because a disproportionate share of the missing data come from colonies in Africa that had both high mortality rates and low economic output. The former African colonies could therefore have driven the correlation between low institutional quality and low economic development. In order to test this possibility to the extent we could, we conducted two simulations.

**Fig 4:**
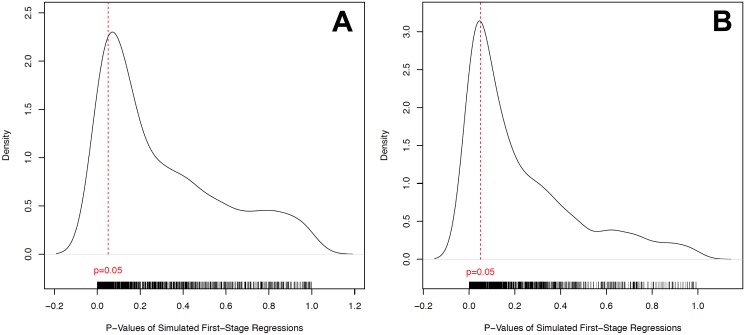
Density plot of P-values using randomly imputed constraint on the executive 1900 values for countries with post-1900 independence dates. The red line indicates a p-value of 0.05. A: Full sample. B: Africa.

In our first, more conservative simulation, we assigned values of 1 for the constraint on the executive in 1900 variable to all of the cases where independence was declared after 1900, other than those cases where values other than 1 were already assigned (i.e. Australia, Egypt, New Zealand, and South Africa). We assumed that since these values were not 1, there were data available for these cases. Rather than assigning all of the other colonies a value of 1, however, we imputed these values by randomly sampling from the colonies for which we had data. We then ran the first stage regressions using these imputed values and calculated a p-value for the mortality instrument. We simulated this process 1,000 times to generate a distribution of p-values. The density of the p-values from these simulations using randomly imputed values for the constraint on the executive measure for countries with post-1900 independence dates are presented in Fig 4 (Panel A). As the density plots show, we fail to obtain significant (at the 5% level) coefficients for the settler mortality instrument in 79.7% of the regressions.

We also ran a second, less conservative simulation where we imputed values for only the African colonies that became independent after 1900. The results of this simulation are presented in Fig 4 (Panel B). Most of the African colonies did not achieve independence until the second half of the 20th century. It is thus unlikely that the data used in the original study relied on good estimates of constraint on the executive in 1900 for most of these cases. Many values were likely assigned rather than measured. It is also true that these countries may be more likely to have lower scores for this variable, given contested boundaries and illegitimate African political institutions in the post-colonial period [4, 6, 26]. Yet, the fact that such places were firmly under the control of democracies like Britain and France means that the executive of these countries ultimately had to answer to democratically elected legislative bodies in the homeland. Thus, executives’ actions could have been more constrained than rulers in recently independent states. Further, we are cautious about assigning low values for the constraint on the executive for former African colonies because settler mortality rates were particularly high there (see Fig 1). It is quite possible that the combination of imputing low values of the quality of political institutions to regions with high settler mortality rates could have created a spurious correlation that favors the institutional hypothesis of economic development. At the least, we would like to test how sensitive the models’ p-values are to this assumption.

In order to perform our second simulation, we took as given the values of the constraint on the executive in 1900 for all colonies other than those in Africa that were not independent in 1900. We then coded the African colonies as having a value of 1 on this variable, per the imputation assumptions in the original dataset, and replaced these for the African colonies by drawing randomly from the distribution of values for other colonies. We then ran the first stage regression of constraint on the executive in 1900 on the logged settler mortality measure and calculated the p-value for the logged mortality coefficient in the model. We repeated this procedure 1,000 times to obtain the full distribution of p-values. Fig 4 (Panel B) reports the distribution of these p-values from the simulation. The results are consistent with our prior finding about the fragility of the causal claims, as 71.4% of the p-values are insignificant at the 5% level under the simulation.

We admit that our simulations are not perfect tests of the data imputation assumptions used in the original study [12]. It is not surprising that we do not get many significant p-values since we are randomly assigning values from the available data for other colonies. Nevertheless, the fact that the mortality instrument fails under such a large fraction of cases demonstrates that we would hesitate to predict differences in institutional quality from European settler mortality rates and to claim a causal relationship between historical institutions in former European colonies and current differences in economic output.

## Conclusion

We examined the validity of the causal relationship between European colonial institutions and contemporary differences in economic development by testing the robustness of the effects to two core assumptions: the treatment of data for the colonies that were not independent in 1900 and the assumptions behind the instrumental variable regressions in the original study [12]. The thesis that extractive political institutions established by European settlers deterred economic development in former European colonies in Asia, Africa, and the Americas has been highly influential in a number of fields including comparative politics and political economy [10–13], and has led to an institutional turn in the literature on economic development. The insitutional hypothesis has emerged as a counter hypothesis to the geography hypothesis of economic development [1, 3, 5–7, 9], claiming instead that political institutions—rather than geographic factors—resulted in the divergent paths of economic development observed in former European colonies around the world. In contrast to the geography hypothesis, institutional economists have argued that past institutions—rather than disease burdens—determined contemporary differences in economic output between developed and developing economies [10, 11]. Specifically, proponents of the institutional hypothesis have advanced causal arguments that European settler mortality determined differences in the quality of political institutions of former European colonies, which subsequently produced differences in economic development. A number of studies have used European settler mortality as an instrument for past institutions [12, 16, 27] as if mortality were uncorrelated with present-day economic output other than through its effect on the quality of political institutions.

We have demonstrated that the causal effect of European colonial institutions on economic development is highly sensitive to changes in three core modeling assumptions used to establish causality: (i) the exclusion restriction, (ii) the as-if random assignment of the instrument, and (iii) the method of imputation of institutional quality for 43 of the 64 former European colonies that were not independent as of 1900. Controlling for differences in the quality of political institutions, we find that European settler mortality directly predicts differences in present-day economic output (a violation of the exclusion restriction for the instrument). We also find that conditioning on the continent of settlement, the instrument fails to predict past institutions at the first stage of the two-stage least squares estimation for economic output. Our results are robust to different measures of institutions and economic performance, and include an important variable omitted in the original study, institutional age.

The original sample used to establish the causal effect of European colonial institutions on differences in economic development in former colonies [12] relied on modeling assumptions that leaned in favor of the proposed negative relationship between settler mortality and institutional quality. Our findings demonstrate that the use of settler mortality rates as an instrument for the quality of political institutions may be inappropriate, because mortality rates varied with differences in mean temperature within continents, and because mortality rates directly predict differences in present-day economic output. Our results that variation in mean temperature explains differences in settler mortality rates and present-day economic output appear to support the geography hypothesis of economic development, which views geography as an important determinant of economic development through its effect on the disease burden of countries [1, 2, 4, 6–9]. Indeed, our findings show that geography may be inextricably linked to human development, contributing to differences in economic productivity through both the quality of political institutions and the prevalence of diseases.
